# Increased Nitrate Intake From Beetroot Juice Over 4 Weeks Changes the Composition of the Oral, But Not the Intestinal Microbiome

**DOI:** 10.1002/mnfr.70156

**Published:** 2025-06-16

**Authors:** Rebeka Fejes, Joana Séneca, Petra Pjevac, Martin Lutnik, Stefan Weisshaar, Nina Pilat, Romy Steiner, Karl‐Heinz Wagner, Richard J. Woodman, Catherine P. Bondonno, Jonathan M. Hodgson, David Berry, Michael Wolzt, Oliver Neubauer

**Affiliations:** ^1^ Department of Nutritional Sciences University of Vienna Vienna Austria; ^2^ Research Platform Active Ageing University of Vienna Vienna Austria; ^3^ Vienna Doctoral School of Pharmaceutical Nutritional and Sport Sciences University of Vienna Vienna Austria; ^4^ Joint Microbiome Facility of the Medical University of Vienna and the University of Vienna Vienna Austria; ^5^ Centre for Microbiology and Environmental Systems Science Department of Microbiology and Ecosystem Science Division of Microbial Ecology University of Vienna Vienna Austria; ^6^ Department of Clinical Pharmacology Medical University of Vienna Vienna Austria; ^7^ Department of Cardiac Surgery Medical University of Vienna Vienna Austria; ^8^ Center for Biomedical Research and Translational Surgery Medical University of Vienna Vienna Austria; ^9^ Department of General Surgery Division of Transplantation Medical University of Vienna Vienna Austria; ^10^ Flinders Centre for Epidemiology and Biostatistics Flinders University Adelaide South Australia Australia; ^11^ Nutrition & Health Innovation Research Institute School of Medical and Health Sciences Royal Perth Hospital Research Foundation Edith Cowan University Joondalup Western Australia Australia; ^12^ Centre for Health Sciences and Medicine University for Continuing Education Krems Krems Austria

**Keywords:** cardiovascular health, dietary nitrate, intestinal microbiome, nitric oxide, oral microbiome

## Abstract

Inorganic dietary nitrate, metabolized through an endogenous pathway involving nitrate reducing bacteria, improves cardiovascular health, but its effects on the oral and intestinal microbiomes of older adults with treated hypertension are unknown. Our study investigated the effects of nitrate from beetroot juice on the oral and intestinal microbiomes of this population. A randomized, double‐blind, placebo‐controlled crossover trial was conducted with 15 participants (age range: 56–71 years), who consumed nitrate‐rich or nitrate‐depleted (placebo) beetroot juice for 4 weeks. The oral microbiome analysis revealed an increase in Neisseria and a decrease in Veillonella relative abundance (for both, PERMANOVA *p* < 0.001), with no significant changes in the intestinal microbiome composition. Our findings suggest that an increased dietary nitrate intake from a vegetable source may selectively modulate the oral microbiome and promote an increased abundance of nitrate‐reducing species, which was previously associated with improved cardiovascular health outcomes.

## Graphical abstract

This randomized, double‐blind, and placebo‐controlled crossover study investigated the effects of an increased intake of inorganic nitrate from beetroot juice over 4 weeks on the oral and intestinal microbial communities in older adults with treated high blood pressure. The findings suggest that dietary nitrate from a vegetable source selectively modulates the oral microbial community and promotes an increased abundance of nitrate‐reducing species in the mouth. This may be important for the generation of the vasoactive signaling molecule nitric oxide (NO) and was previously associated with improved cardiovascular health outcomes.

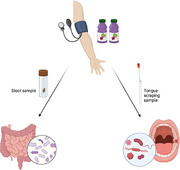

**Abbreviations**
3H POST: 3 h after the intake of the first dose of beetroot juice
4WK POST: 4 weeks after beetroot juice supplementation
ASVs: amplicon sequence variants
CVD: cardiovascular diseases
FBF: forearm blood flow
GSH/GSSG: reduced to oxidized glutathione ratio
hsCRP: high sensitivity C‐reactive protein
ICAM‐1: intercellular adhesion molecule 1
IL‐10: interleukin‐10
IL‐6: interleukin‐6
MCP‐1: monocyte chemoattractant protein‐1
NO: nitric oxide
oxLDL: oxidized low‐density lipoprotein
PRE: before beetroot juice supplementation
TNF‐α: tumor necrosis factor
TNFRSF1A: tumor necrosis factor receptor superfamily member 1A
TNFRSF1B: tumor necrosis factor receptor superfamily member 1B
VCAM‐1: vascular cell adhesion molecule 1

## 1 Introduction

Cardiovascular diseases (CVD) continue to be the primary cause of morbidity, disability, and mortality in modern societies [1, 2] and the risk of experiencing CVD rises with increases in blood pressure [2, 3]. The composition of one's diet can significantly affect health. Observational studies revealed that diets abundant in vegetables are linked to a lowered risk of CVD [4, 5]. Green leafy vegetables and beetroot are particularly rich sources of inorganic nitrate. A growing body of evidence indicates that the intake of dietary nitrate via nitric oxide (NO) offers various advantageous effects on cardiovascular health [6, 7, 8]. Nitric oxide is a signaling molecule crucial for vascular function, because it regulates vascular blood flow, the vascular tone, and maintains vascular integrity [7, 9, 10]. Endogenously, NO is produced via the L‐arginine‐synthase pathway. The availability of NO in the vasculature declines with advancing age due to oxidative stress, chronic low‐grade inflammation, higher NO degradation rates, and lower NO production rates [7, 8, 10]. Nitric oxide produced via this pathway is metabolized into nitrate and nitrite. These compounds are recycled back to NO through a nitrate‐nitrite‐NO pathway. Nitrate from the diet can increase NO through this pathway. The nitrate‐nitrite‐NO pathway begins with the ingestion of dietary nitrate, which is absorbed in the small intestine and enters the bloodstream. A part of the bioavailable nitrate—approximately 25%—is concentrated in saliva, where it gets reduced to nitrite by oral nitrate reducing bacteria [11] such as *Actinomyces*, *Veillonella*, and *Neisseria* [12]. Nitrate‐reducing bacteria are found predominantly in the dorsal clefts of the tongue [13]. When saliva is swallowed, nitrite is further converted to NO in the acidic environment of the stomach or by enzymatic action in tissues and blood [6].

In addition to the contribution of the oral microbiome to the generation of the vasoactive signaling molecule NO in the nitrate‐nitrite‐NO pathway, an increasing body of evidence suggests that the gut microbiome plays an important role for cardiovascular health [14]. Findings from a recent study suggest that a dysbiosis of the gut microbiome contributes to age‐related arterial dysfunction, which highlights its potential as a target for therapies aimed at maintaining arterial health with advancing age and potentially lowering the risk of CVD [15]. Changes in the gut microbiota are linked to CVD, with experimental evidence showing that metabolites like trimethylamine N‐oxide and phenylacetylglutamine can influence CVD through specific pathways and receptors [16]. Intestinal bacteria also reduce nitrate to nitrite, however, the knowledge of the effects of an increased intake of dietary nitrate on the human intestinal microbiome is limited.

With this randomized, double‐blind, placebo‐controlled crossover study, we addressed the need to investigate whether an intervention with dietary nitrate affects the oral and intestinal microbiomes in older adults with hypertension, as an important clinical population at an increased CVD risk. The pre‐defined primary endpoints of the study were vascular function, assessed by measuring forearm blood flow (FBF) responses to acetylcholine, and 24‐h ambulatory systolic blood pressure before and after the 4‐week treatments with nitrate‐rich versus nitrate‐depleted (placebo) beetroot juice. The outcomes of these and other endpoints (including blood biomarkers of oxidative stress and inflammation) have been reported previously [17, 18]. As reported herein, another important aim of this study was to assess whether the oral and gut microbial communities change with an increased intake of inorganic nitrate from beetroot juice over 4 weeks. In parallel, we investigated whether the baseline composition of and alterations in oral and gut microbiomes were correlated with nitrate and nitrite levels in blood and saliva, blood biomarkers of oxidative stress and inflammation, and various blood pressure measures including 24‐h ambulatory blood pressure.

## 2 Experimental Section

### 2.1 Ethical Statement

Approval for the study was obtained from the Medical University of Vienna's Ethics Committee (ethics number: EK 2238/2018, 01/15/2019), and the trial was registered on ClinicalTrials.gov (NCT04584372).

### 2.2 Study Design

**FIGURE 1:**
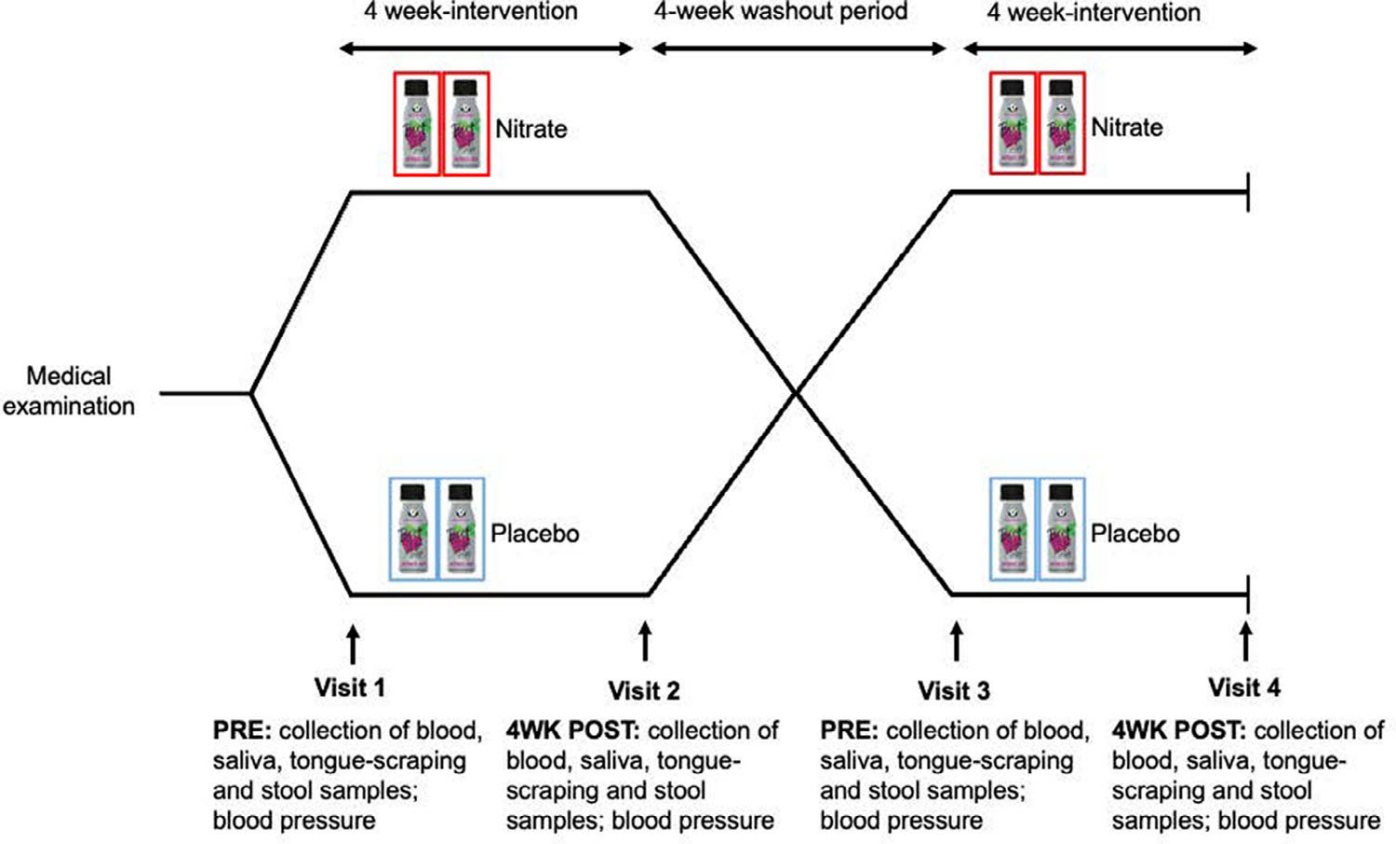
Study design. Adapted from. [17]

We employed a randomized, double‐blind, and placebo‐controlled crossover study, with two 4‐week treatment phases divided by a 4‐week washout period. These phases consisted of a 4‐week intervention with nitrate‐rich beetroot juice (Nitrate) and a 4‐week intervention with nitrate‐depleted beetroot juice (Placebo). Participants adhered to a low‐nitrate diet and maintained their usual lifestyle and medication regimen throughout the study. As mentioned above and reported previously [17], the primary endpoints of the study were vascular function assessed through FBF responses to acetylcholine, and 24‐h ambulatory systolic blood pressure, both before and after the 4‐week treatment periods (Nitrate vs. Placebo). The study design and the time‐points of sample collection are summarized in Figure 1.

### 2.3 Study Participants

This study involved a group of 15 men and women, aged between 56 and 71, who were diagnosed with hypertension and were under treatment with anti‐hypertensive medication. Out of the initial 409 individuals screened for participation, 20 were randomized, and ultimately 15 completed the study, as previously reported [17]. Detailed information regarding the recruitment process and the baseline characteristics of these 15 participants has been outlined in an earlier publication [17]. Before joining the study, each participant provided written consent after undergoing a thorough medical examination and completed validated questionnaires on dietary habits [19] and physical activity levels [20] to establish baseline data. An exclusion criterion was the use of antibacterial mouthwash 4 weeks prior to randomization and throughout the study, which could affect the oral commensal microbiome and disrupt the nitrate–nitrite–NO pathway. The complete list of inclusion and exclusion criteria was previously published alongside the primary outcomes of the study [17].

### 2.4 Dosage Information

After successful eligibility examination, the participants were enrolled and randomly allocated to one of two treatment groups. Both the participants and the investigators remained blinded to the treatment assignments throughout the study. The two treatments consisted of: (1) consuming two 70 mL bottles of nitrate‐rich (Nitrate) beetroot juice daily (12.9 mmol, i.e., ∼800 mg of nitrate); or (2) consuming two 70 mL bottles of nitrate‐depleted (Placebo) beetroot juice as a placebo (≤0.04 mmol of nitrate), both over a 4‐week period. All products were sourced from the same supplier (Beet It, James White Drinks Ltd., UK). Participants were instructed to maintain to their normal lifestyle (including medication, diet, and physical activity) but reduce the intake of nitrate‐rich foods, with a list of what to avoid or limit. The compliance was assessed at every study visit with standard questionnaires, for physical activity [20] and the diet [19], adapted for nitrate‐rich foods.

### 2.5 Oral and Intestinal Microbiome Sample Collection

Samples for assessing oral and intestinal microbiome composition were collected before (PRE) and 4 weeks after (4WK POST) nitrate and placebo beetroot juice supplementation. Tongue scraping samples were collected for oral microbiome measurement by gently scraping the surface of the tongue with a swab (COPAN 480CE eSwab with 1 mL Amies medium) a few times to capture nitrate‐reducing bacteria located in the deep clefts of the tongue [13]. To examine the composition of the intestinal microbiome, participants were provided with stool catchers and instructed to collect a stool sample within 24 h before their study visit, which they brought to the clinic. All samples were frozen and stored at −80°C until further analysis.

### 2.6 Oral and Intestinal Microbiome Characterization

DNA was extracted from stool and tongue samples using the Fast DNA Stool Mini kit (QIAamp) and the Microbiome DNA isolation kit (QIAamp), respectively, following each kit manufacturer's instructions. The V4 hypervariable region of the bacterial and archaeal 16S rRNA gene was amplified using primers 515F/806R [21, 22] and subsequently prepared for sequencing following a dual‐step barcoding approach, as described previously [23], and sequenced on an Illumina MiSeq (2 × 300 bp). DNA extraction, sequencing, and raw data processing was performed at the Joint Microbiome Facility of the Medical University of Vienna and the University of Vienna (project ID JMF‐2303‐03). Amplicon pools were extracted from the raw sequencing data using the FASTQ workflow in BaseSpace (Illumina) with default parameters. Demultiplexing was performed with the python package demultiplex (Laros JFJ, github.com/jfjlaros/demultiplex) allowing one mismatch for barcodes and two mismatches for linkers and primers. Amplicon sequence variants (ASVs) were inferred using the DADA2 R package v1.42 [24] applying the recommended workflow [25]. FASTQ reads 1 and 2 were trimmed at 220 nt and 150 nt with allowed expected errors of 2. ASV sequences were subsequently classified using DADA2 and the SILVA database SSU Ref NR 99 release 138.1 [26, 27] using a confidence threshold of 0.5. ASVs without classification or classified as eukaryotes, mitochondria, or chloroplasts, as well as well‐known buffer contaminations were removed. After filtering, only samples with at least 1000 read pairs were kept for further analyses. Downstream analyses were performed using R v4.3.2 and Bioconductor v3.16 packages SummarizedExperiment v1.32, SingleCellExperiment v1.24, TreeSummarizedExperiment v2.8 [28], mia v1.8, vegan v2.6‐4 [29], phyloseq v1.44 [30], microbiome v1.22, microViz v0.10.8 [31], and ALDEx2 v1.32 [32]. Alpha diversity (i.e., richness and diversity indexes) was calculated on rarified data (4762 read pairs/sample) using R packages vegan and mia. Beta diversity was calculated by performing a PCoA using the Aitchison distance, with R package microViz. The difference in per‐group centroids was tested with a PERMANOVA on Aitchison distances using R packages vegan and microViz. Pairwise differential abundance testing between conditions was performed using DESeq2 with alpha = 0.05 and otherwise default parameters after adding a pseudocount of 1 to the data.

### 2.7 Correlation Analysis of the Top‐Most Abundant Oral and Intestinal Bacterial Genera With Blood Pressure Measures and Biochemical Variables

Correlations between centered log ratio transformed counts of the top‐most abundant genera were conducted against blood pressure measures and biochemical variables for both datasets using ALDEx2's correlation test using the Spearman's correlation. Correlations with a corrected *p* value after with FDR multiple testing correction were deemed significant. The following clinical and biochemical parameters were included in the analysis in association with the composition of the microbiomes: ambulatory 24‐h blood pressure; blood plasma and salivary nitrate and nitrite; low‐density lipoprotein (LDL), oxidized LDL (oxLDL), reduced and oxidized glutathione ratio (GSH/GSSG), f_2_‐isoprostanes, high‐sensitivity C‐reactive protein (hsCRP), monocyte chemoattractant protein‐1 (MCP‐1), E‐selectin, intercellular adhesion molecule 1 (ICAM‐1), interleukin‐10 (IL‐10), interleukin‐6 (IL‐6), P‐selectin, tumor necrosis factor receptor superfamily member 1A (TNFRSF1A), tumor necrosis factor receptor superfamily member 1B (TNFRSF1B), tumor necrosis factor (TNF‐α), vascular cell adhesion molecule 1 (VCAM‐1), proportion of classical, non‐classical and intermediate monocyte subsets. Detailed information on the assessment methods and the outcomes of these variables have been reported in previous publications [17, 18]. Correlations between the top 50 most abundant oral bacterial genera and measured parameters in our study population are summarized in Table S1.

### 2.8 Availability

The 16S rRNA gene amplicon sequencing data has been deposited at the Sequence Read Archive under the BioProject accession PRJNA1152522.

## 3 Results

### 3.1 Oral Microbiome Profiles

**FIGURE 2:**
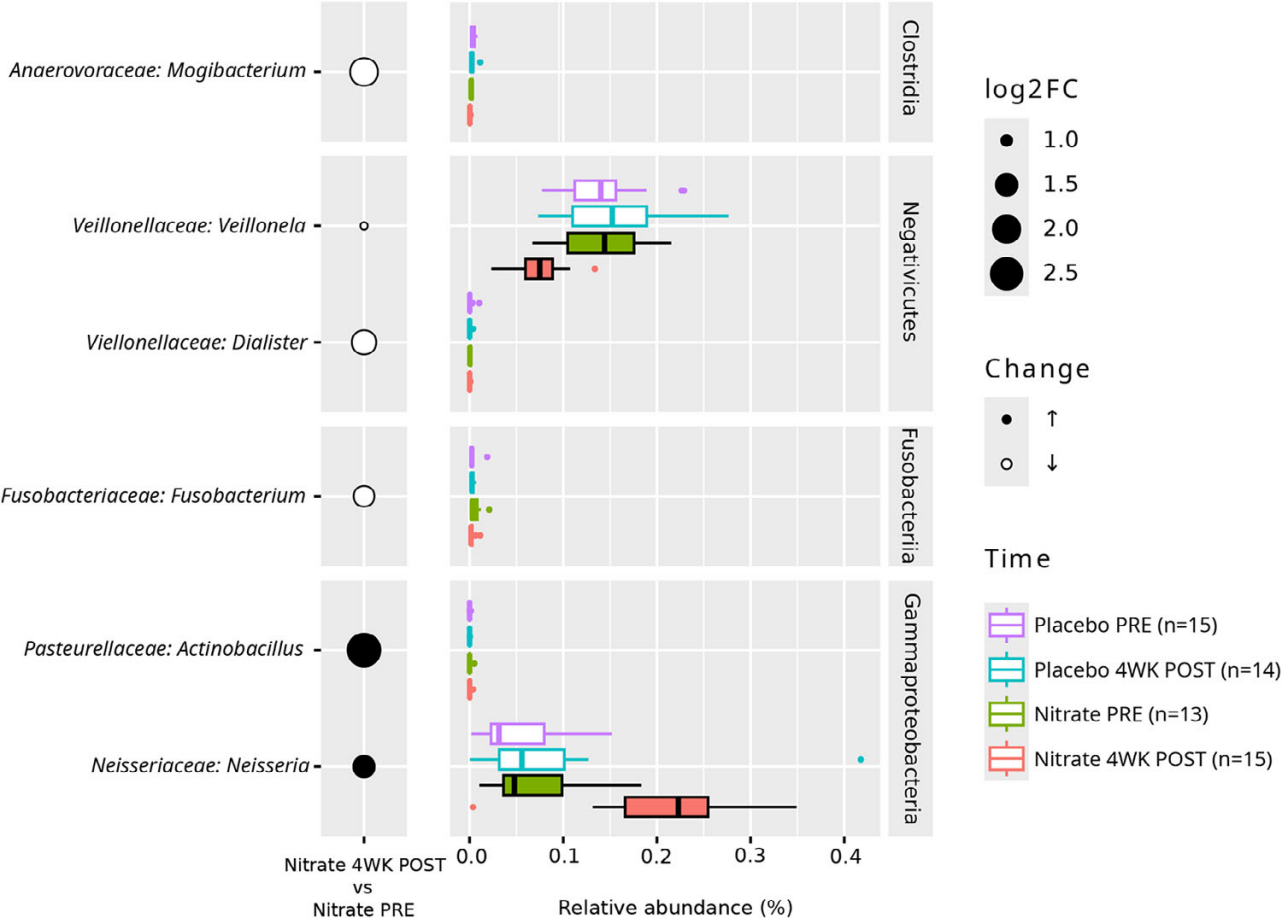
Differentially abundant oral bacterial genera found between before (PRE) and 4 weeks after (4WK POST) beetroot juice administration in placebo and nitrate treatments. Statistically significant differential abundance bacterial genera were only found in the Nitrate treatment (filled boxplots). The circles represent the Log2fold change of bacterial genera between 4WK POST compared to before (PRE) the treatments, and the color of the circle represents an increase or decrease. The barplots indicate the relative abundance of bacterial genera on all treatments. The number of biological replicates per condition is shown next to the color legend. The boxplots depict the median (Q2) as a line in the middle and the inter quartile range (IQR, Q1 and Q3) in the bottom and top, respectively. The whiskers depict the distance up to 1.5 x the IQR. Data points larger than this value are considered outliers and shown as individual dots.

**FIGURE 3:**
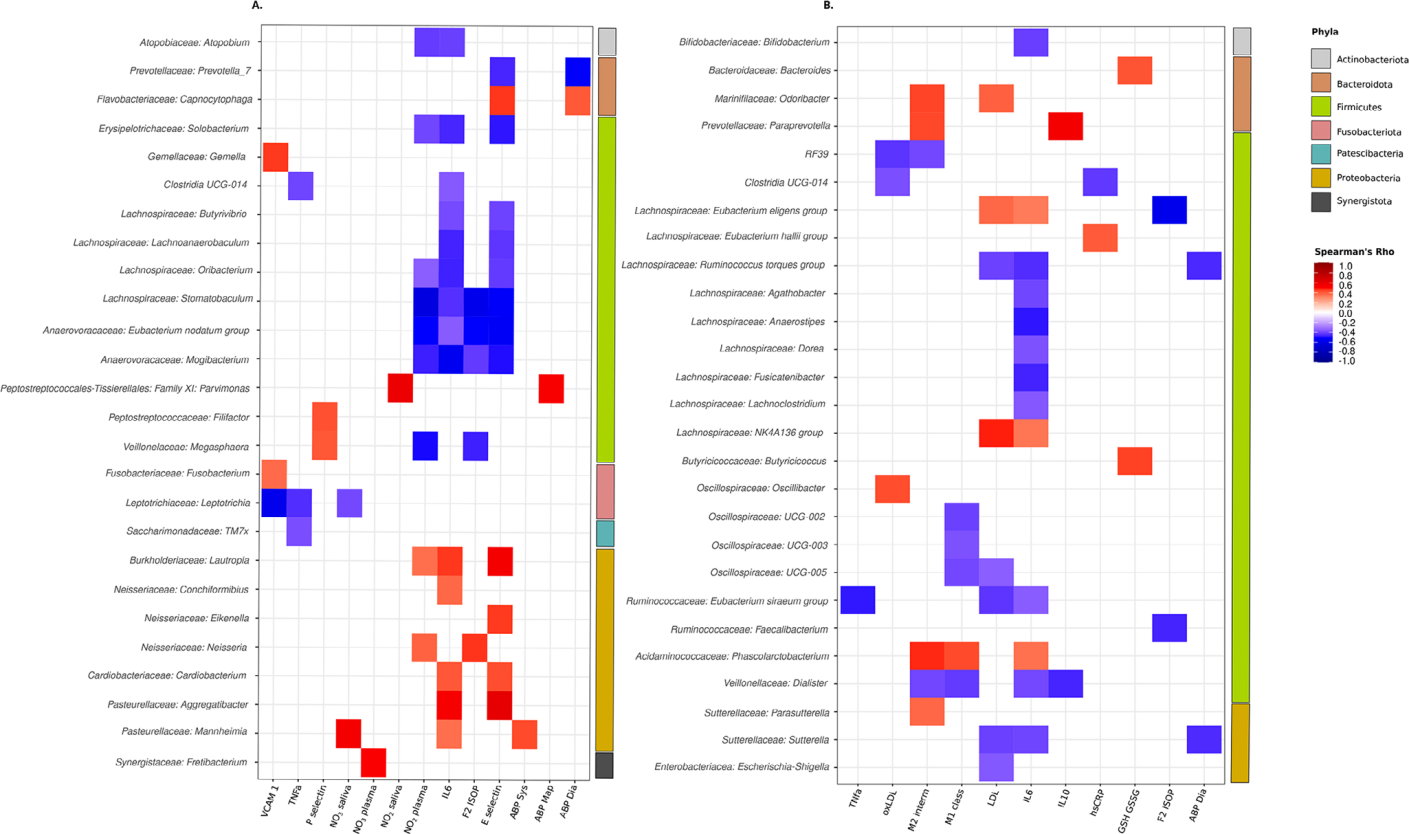
Correlations between the top‐most abundant oral and intestinal bacterial genera and measured clinical and biochemical parameters in our study population of 15 participants with grade 1 hypertension. Panel A shows correlations between the top 50 most abundant oral bacterial genera and parameters. Panel B shows correlations between the top 50 most abundant bacterial genera in the stool microbiome and parameters. Abbreviations: ABP Dia = diastolic ambulatory 24 h blood pressure, ABP Map: ambulatory mean arterial ambulatory 24 h blood pressure, ABP Sys = systolic ambulatory 24 h blood pressure, E selectin = E‐selectin, F2 ISOP = f2‐isoprostanes, GSH_GSSG = reduced to oxidized glutathione ratio, hsCRP = high sensitive c‐reactive protein, IL6 = interleukin‐6, IL10 = interleukin‐10, LDL = low‐density lipoprotein, M1_class = classical monocytes, M2_intern = intermediate monocytes, NO2 plasma = plasma nitrite concentration, NO2 saliva = salivary nitrite concentration, NO3 plasma = plasma nitrate concentration, NO3 saliva = salivary nitrate concentration, oxLDL = oxidized low‐density lipoprotein, P selectin = P‐selectin, TNFa = tumor necrosis factor alpha, VCAM 1 = vascular cell adhesion molecule 1.

After confirming no significant difference in baseline (PRE) microbiome samples between the nitrate and placebo groups (Figure S1), we tested if the increased intake of nitrate through beetroot juice produced significant changes in oral microbial communities. While no significant differences were observed in species richness and evenness (alpha diversity), between groups before (PRE) or after (POST) supplementation, regardless of supplementation type (Nitrate vs. Placebo) (Figure S2), pairwise comparisons revealed significant differences in oral microbial community composition (beta diversity) only between PRE Nitrate and POST Nitrate supplementation (PERMANOVA: *p* < 0.001, Beta dispersions ANOVA: *p* > 0.05). Differential abundance testing showed significant changes in relative abundance of several bacterial genera in the oral microbiome of participants 4 weeks after beetroot juice intake (4WK POST Nitrate) compared to baseline (PRE Nitrate) (Figure 2), while no significant differences were detected in individuals after receiving placebo supplementation (4WK POST Placebo vs. PRE Placebo). The relative abundance of genus Neisseria increased, while the relative abundance of genus *Veillonella* decreased after supplementation. Correlations between the top 50 most abundant oral and intestinal bacterial genera and blood pressure measures and biochemical variables are visualized in Figure 3.

### 3.2 Stool Microbiome Profiles

We detected no significant differences in the intestinal microbial community structure with nitrate supplementation. Furthermore, correlations between the top‐most abundant bacterial genera and measured parameters did not reveal any distinct effects that may be attributable to the study intervention (Figure 3).

## 4 Discussion

In the currently reported randomized, placebo‐controlled crossover study, we aimed to assess whether the oral and gut microbial composition change with a 4‐week long intervention with dietary nitrate from concentrated beetroot juice. Furthermore, we assessed if the baseline composition of the oral and intestinal microbiomes and changes therein were correlated with blood and salivary nitrate and nitrite levels, blood biomarkers of oxidative stress and inflammatory, and blood pressure. The data of our study suggest that an increased nitrate intake from beetroot juice over 4 weeks changes the oral, but not the intestinal microbiome. The relative abundance of the genus Neisseria significantly increased, with a significant decrease in Veillonella relative abundance at the same time. The stool microbiome remained unchanged after supplementation. The lack of changes to intestinal microbiome composition can be considered as positive outcome, since changes in NO metabolism have previously been observed to be associated with increased inflammation in the intestine [33, 34, 35].

The oral microbiome's obligatory role is underlined by the findings of the various studies investigating the effect of antibacterial mouthwashes on NO metabolism and blood pressure. Studies intervening with antibacterial mouthwashes observed a disruption in the nitrate–nitrite–NO pathway. Rats given mouthwash with nitrate had fewer nitrate‐reducing bacteria, lowering gastric nitric oxide, and circulating nitrite levels. Additionally, this reduced the protective effect against ulcers and prevented the decrease in blood pressure associated with nitrate supplementation [36]. In a study with seven healthy volunteers, using a chlorhexidine‐containing mouthwash before consuming sodium nitrate did not change nitrate levels in saliva or plasma, but it inhibited nitrate conversion to nitrite in saliva and reduced plasma nitrite increase [37]. Finally, in a randomized crossover study, 15 individuals with treated hypertension were given antibacterial mouthwash for 3 days, while a control group used water. The mouthwash disrupted the nitrate–nitrite–NO pathway, resulting in an increase in systolic blood pressure [38].

The oral microbiome contains several microbial groups capable of reducing nitrate. Among those, members of the genus *Veillonela* have been shown to be the most prevalent nitrate‐reducers, followed by *Actinomyces*, *Rothia*, and *Staphylococcus* spp. [13]. The findings of Hyde et al. also confirmed the *Veillonella* spp. as the most abundant nitrate‐reducing species, however, *Prevotella*, *Neisseria*, and *Haemophilus* were detected in higher abundance than the *Actinomyces* spp. [6, 39].

In a clinical context, lower abundances of Veillonela have been linked to hypertension. According to novel findings, hypertensive women demonstrated a lower abundance of Veillonella bacteria and impaired oral nitrate reduction—higher nitrate reductase activity was associated with lower baseline blood pressure—compared to normotensive women [40]. Higher oral nitrate reductase activity was linked to lower initial diastolic blood pressure and stronger reductions after dietary nitrate supplementation in women [40]. Furthermore, an additional study found that daily treatment with 250 mL nitrate‐rich beetroot juice changed the salivary microbiome composition, with *Rothia mucilaginosa* showing increasing tendency and *Neisseria flavescens* increased significantly after nitrate supplementation compared to the placebo treatment [41]. Vanhatalo et al. investigated the modulating effect of microbiome on NO homeostasis [42]. Compared to placebo—nitrate‐depleted beetroot juice—daily 140 mL nitrate‐rich beetroot juice supplementation increased the abundance of Rothia and Neisseria by 127% and 351%, respectively, while decreasing the abundance of *Prevotella* and *Veillonella* by 60% and 65%, respectively [42]. Finally, Jockel‐Schneider and colleagues examined the changes in the oral microbiome composition in periodontal recall patients after daily 300 mL nitrate‐rich vegetable juice consumption [43]. Following the nitrate‐based intervention there was a significant increase in *Rothia* and *Neisseria*, including species that reduce nitrate, but no changes were observed in the control group [43].

The findings of our clinical study show an increased abundance of *Neisseria* spp. after 4 weeks of intervention with the nitrate‐rich beetroot juice, and interestingly, the abundance of *Veillonella* spp. declined, while no significant changes in other nitrate‐reducing bacteria species were detected. In the oral microbiome of our participants, *Neisseria* spp. and *Veillonella* spp. were the most abundant species. The decrease of Veillonella with the simultaneous increase of Neisseria is in line with previous reports from literature, that dietary nitrate supplementation promotes the increase in abundance of denitrifying species—which is associated with improved cardiometabolic outcomes [44]. Additionally, regarding nitrate‐reducing species, we observed a significant positive correlation between Neisseria and plasma nitrate (*r* = 0.46) and plasma nitrite (*r* = 0.39). These correlations support the aforementioned properties of an increased dietary nitrate intake on the oral microbiome. As we have reported previously [17], the increase in plasma and salivary nitrate and nitrate increased the potential for an augmented NO formation. Whereas the increase in plasma and saliva nitrate and nitrite did not translate into sustained improvements in vascular function and blood pressure in the participants of our study [17], our data suggest a subtle change toward a less prooxidative profile following the nitrate intervention [18]. Furthermore, we observed a decrease in plasma hsCRP levels following the daily consumption of nitrate‐rich beetroot juice over 4 weeks, as compared with baseline [18]. These findings offer tentative support for additional mechanisms through which an increased intake of nitrate from plant sources elicits cardiovascular benefits. The data as reported herein agree with the concept that nitrate‐sensitive and ‐reducing oral bacteria play a central role in the modulation of these beneficial effects.

As another noteworthy aspect, emerging data suggest that an increased intake of dietary nitrate may positively modulate human health through effects on oral health [45]. It is increasingly recognized that oral health has an important impact on systemic health, including cardiovascular health [6, 45]. The results of a recent systematic review by Alhulaefi et al. indicate that modulating the oral microbiome, decreasing salivary pH and gingival inflammation through dietary interventions with nitrate might favorably affect markers of systemic health [45]. The current study does not enable firm conclusions about possible effects on oral health. However, we observed similar changes in the oral microbiome composition as in previous randomized controlled trials that reported benefits on oral health markers (such as an increase in the relative abundance of Neisseria) [45]. It may therefore be speculated that the observed shift in the oral microbiome of our study participants, potentially, may have had a similar beneficial effect on oral health markers.

Despite numerous studies investigating the health effect of dietary nitrate, the effects of dietary nitrate on the microbiota of the lower gastrointestinal tract are still poorly understood [46]. One study investigating the effects of nitrate and nitrite from processed meat and drinking water on the intestinal microbiome found no differences in microbial richness, diversity, and community structure following exposure to elevated nitrate and nitrite concentrations [47]. Another recent study examined the modulation of gut microbiota after 2 weeks of beetroot juice consumption, which contained ca. 228.5 mg nitrate per portion [48]. Relative to baseline, transient alterations in the abundance of some taxa were observed, for example, *Romboutsia* and *Christensenella*, and an increase in *Akkermansia muciniphila* and a decline in Bacteroides fragilis were noted after 3 days of drinking beetroot juice, with their abundances returning to baseline abundances after 14 days [48].

In our study, we detected no differentially abundant bacterial genera following a 4‐week nitrate supplementation with beetroot juice in gut samples of individuals with diagnosed hypertension, suggesting that the intestinal microbiome's metabolic potential for NO metabolism has not majorly been disrupted. In summary, our trial showed that increased nitrate intake from beetroot juice changed the composition of the oral microbiome, specifically increasing Neisseria spp. and decreasing *Veillonella* spp., which may be associated with improved cardiometabolic outcomes. The stability of the intestinal microbiome may be advantageous, as it avoids potential disruptions in NO metabolism linked to inflammatory bowel disease [33, 34, 35], highlighting the potential targeted and safe modulation of the oral microbiome by dietary nitrate intake.

Together, the findings from this study provide new information on the efficacy of inorganic nitrate in the form of beetroot juice to selectively modulate the oral microbial community, without eliciting changes in the intestinal microbiome. Future investigations may especially focus on whether dietary nitrate from plant sources modulates the composition of the oral microbiome in a dose‐dependent manner and whether potential dose‐dependent effects are linked with more or less pronounced clinical outcomes.

This research was funded by the Austrian Science Fund (FWF) within the Clinical Research (KLIF) program, granted to Oliver Neubauer (FWF project number: KLI 858). This grant also supported the salaries of Oliver Neubauer (partially) and Rebeka Fejes (completely). At the Medical University of Vienna, we thank study nurses Carola Fuchs and Claudia Eder for their organisational assistance. We also gratefully thank all the study participants for their time and efforts in participating in this study. The computational results of this work have been achieved using the Life Science Compute Cluster (LiSC) of the University of Vienna.

Open access funding provided by Universitat Wien/KEMO.

## Conflicts of Interest

The authors declare no conflicts of interest.

## Supporting information




**Supporting File 1**: mnfr70156‐sup‐0001‐SuppMat.docx.

## Data Availability

Data openly available in a public repository that issues datasets with DOIs.
