# Supplementary material for: Increased Nitrate Intake From Beetroot Juice Over 4 Weeks Changes the Composition of the Oral, But Not the Intestinal Microbiome
Source: Mol Nutr Food Res. 2025 Jun 16;69(20):e70156. doi: 10.1002/mnfr.70156 (PMC12538535; doi:10.1002/mnfr.70156)

**Supplementary Table 1**: Correlations between the top 50 most abundant oral bacterial genera and measured clinical and biochemical parameters in our study population of 15 participants with grade 1 hypertension

| Genus | Variable | Correlation coefficient | P value |
| --- | --- | --- | --- |
| Eubacterium nodatum group | Saliva nitrate | -0.53 | 0.0011 |
|  | Plasma nitrate | -0.51 | 0.0018 |
|  | Plasma nitrite | -0.50 | 0.0021 |
|  | Saliva nitrite | -0.36 | 0.0417 |
| Aggregatibacter | Saliva nitrate | 0.60 | 0.0003 |
|  | Saliva nitrite | 0.54 | 0.0019 |
| Atopobium | Plasma nitrite | -0.42 | 0.0128 |
|  | Saliva nitrite | -0.41 | 0.0159 |
| Butyrivibrio | Saliva nitrate | -0.40 | 0.0311 |
|  | Saliva nitrite | -0.39 | 0.0365 |
| Capnocytophaga | Saliva nitrate | 0.46 | 0.0045 |
|  | VCAM1 | 0.40 | 0.0423 |
| Cardiobacterium | Saliva nitrate | 0.42 | 0.0157 |
|  | Saliva nitrite | 0.41 | 0.0239 |
| Conchiformibius | Saliva nitrite | 0.37 | 0.0414 |
| Eikenella | Saliva nitrate | 0.46 | 0.0065 |
| Filifactor | ABP-SYS | 0.42 | 0.0471 |
| Fretibacterium | f2-isoprostanes | 0.52 | 0.0039 |
| Fusobacterium | ABP-DIA | 0.37 | 0.0480 |
| Gemella | ABP-DIA | 0.46 | 0.0107 |
| Lachnoanaerobaculum | Saliva nitrite | -0.46 | 0.0067 |
|  | Saliva nitrate | -0.43 | 0.0092 |
| Lautropia | Saliva nitrate | 0.55 | 0.0011 |
|  | Saliva nitrite | 0.46 | 0.0087 |
|  | Plasma nitrite | 0.36 | 0.0439 |
| Leptotrichia | ABP-DIA | -0.58 | 0.0002 |
|  | ABP-MAP | -0.45 | 0.0243 |
|  | Eselectin | -0.39 | 0.0475 |

Continued on the next page

**Supplementary Table 1** – continued:

| Mannheimia | Eselectin | 0.54 | 0.0012 |
| --- | --- | --- | --- |
|  | Saliva nitrite | 0.36 | 0.0388 |
|  | Pselectin | 0.43 | 0.0425 |
| Megasphaera | Plasma nitrite | -0.49 | 0.0025 |
|  | Plasma nitrate | -0.47 | 0.0049 |
|  | ABP-SYS | 0.41 | 0.0472 |
| Mogibacterium | Saliva nitrite | -0.56 | 0.0013 |
|  | Saliva nitrate | -0.49 | 0.0029 |
|  | Plasma nitrite | -0.47 | 0.0044 |
|  | Plasma nitrate | -0.42 | 0.0144 |
| Neisseria | Plasma nitrate | 0.46 | 0.0050 |
|  | Plasma nitrite | 0.39 | 0.0231 |
| Oribacterium | Saliva nitrite | -0.47 | 0.0063 |
|  | Saliva nitrate | -0.42 | 0.0100 |
|  | Plasma nitrite | -0.35 | 0.0450 |
| Parvimonas | IL-6 | 0.57 | 0.0002 |
|  | TNFa | 0.53 | 0.0014 |
| Prevotella_7 | VCAM1 | -0.52 | 0.0023 |
|  | Saliva nitrate | -0.46 | 0.0045 |
| Solobacterium | Saliva nitrate | -0.48 | 0.0032 |
|  | Saliva nitrite | -0.46 | 0.0072 |
|  | Plasma nitrite | -0.40 | 0,0194 |
| Stomatobaculum | Plasma nitrite | -0.62 | 0.0000 |
|  | Plasma nitrate | -0.58 | 0.0002 |
|  | Saliva nitrate | -0.52 | 0.0014 |
|  | Saliva nitrite | -0.44 | 0.0091 |
| TM7x | ABP-MAP | -0.38 | 0.0499 |
| Clostridia UCG-014 | ABP-MAP | -0.40 | 0.0395 |
|  | Saliva nitrite | -0.36 | 0.0414 |

Abbreviations: ABP-DIA=diastolic ambulatory 24h blood pressure, ABP-MAP: ambulatory mean arterial ambulatory 24h blood pressure, ABP-SYS=systolic ambulatory 24h blood pressure, IL-6=interleukin-6, VCAM1=vascular cell adhesion molecule 1


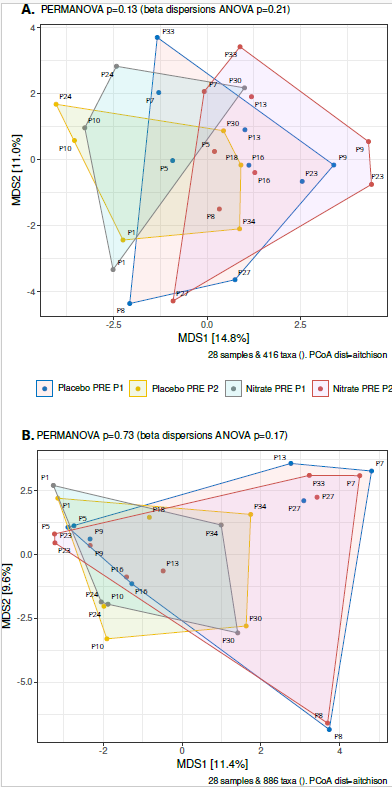


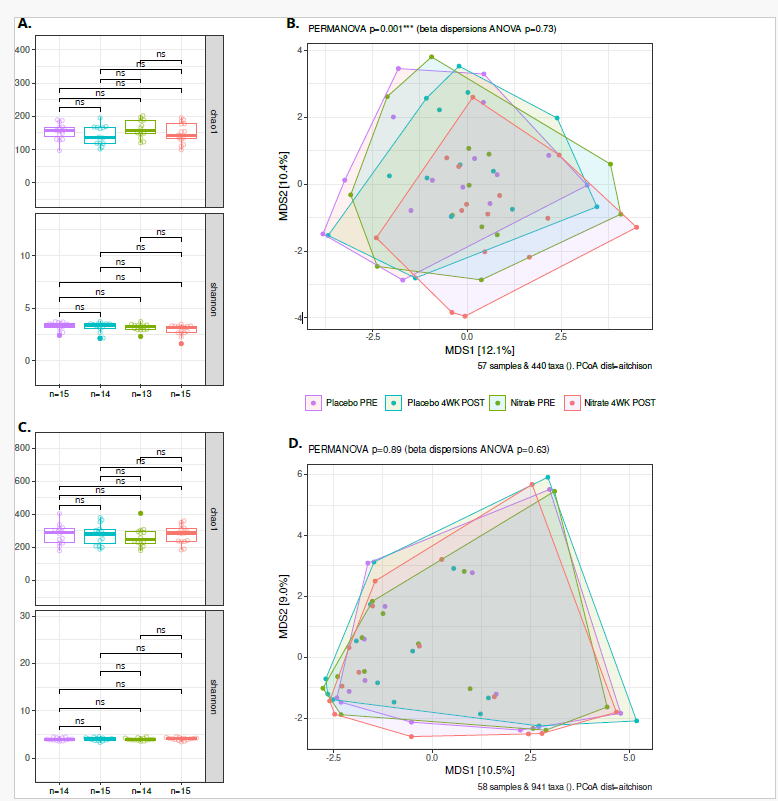

Supplement: Supplementary file 1 — Supporting File 1: mnfr70156‐sup‐0001‐SuppMat.docx. [file MNFR-69-e70156-s001.docx]
